# THE EFFECT OF LIFESPAN TRAUMA EXPOSURE ON PATHOLOGICAL PERSONALITY TRAITS

**DOI:** 10.1093/geroni/igad104.3026

**Published:** 2023-12-21

**Authors:** Lisa Stone-Bury, Julie Hurd, Daniel Segal

**Affiliations:** University of Colorado at Colorado Springs, Colorado Springs, Colorado, United States; University of Colorado at Colorado Springs, Colorado Springs, Colorado, United States; University of Colorado at Colorado Springs, Colorado Springs, Colorado, United States

## Abstract

**Introduction:**

The Alternative Model of Personality Disorders (AMPD) is a dimensional model of personality disorder pathology focusing on pathological personality traits, with five trait domains. Limited knowledge exists about relationships between cumulative exposure to traumatic events and the AMPD, a gap particularly relevant for older adults who have had a lifetime of potential exposure. Method: Older adults (n = 200) completed the Trauma Health Questionnaire (THQ) and Personality Inventory for DSM-5 (PID-5).

**Results:**

Multivariate analysis of covariance (MANCOVA) revealed significant differences in the five PID-5 domains across four levels of cumulative trauma exposure (Pillai’s Trace =.14, F(5, 519) =1.73, p =.04, ηp2=.05). Years since first traumatic event was not significant as a covariate (Pillai’s Trace =.02, F(4, 173) =0.80, p =.55, ηp2=.02). Significant group differences were found in the PID-5’s Psychoticism (F(3, 175) = 5.71, p < .001, ηp2 = .09) and Detachment (F(3, 175) = 2.91, p = .03, ηp2 = .05). According to Bonferroni post-hoc tests, the high trauma exposure group was significantly higher in Psychoticism than low trauma exposure, low to medium trauma exposure, and medium to high trauma exposure groups. For Detachment, the high trauma exposure group was significantly higher than the low trauma exposure and low to medium exposure groups.

**Discussion:**

Findings suggest that cumulative trauma exposure is associated with specific pathological personality traits in older adults. High levels of trauma exposure may be particularly related to eccentric and detached traits that persist in later life, reflecting the need for clinical assessment in both domains.

